# Assessment the reliability of ultrasonography in the imaging of the plantar fascia: a comparative study

**DOI:** 10.1186/s12880-019-0361-1

**Published:** 2019-08-07

**Authors:** Jing Wu, Yuan-zhi Zhang, Yang Gao, Tian-you Luo

**Affiliations:** 1 Department of Radiology, The Affiliated Hospital of Inner Mongolia Medical University, Hohhot, People’s Republic of China; 2 Department of Orthopaedics, The Affiliated Hospital of Inner Mongolia Medical University, No 1, Tongdao North Road, Huimin District, 010059 Hohhot, People’s Republic of China; 3 Department of MR, The Affiliated Hospital of Inner Mongolia Medical University, Hohhot, People’s Republic of China; 4 Department of Radiology, The First Affiliated Hospital of Chongqing Medical University, Chongqing, People’s Republic of China

**Keywords:** Ultrasound, Plantar fascia, Reliability

## Abstract

**Background:**

Imaging methods for the plantar fascia have included radiography, ultrasound and magnetic resonance imaging (MRI), all of which have provided valuable information. This study assessed the reliability of ultrasonography examinations of the plantar fascia using a comparative study.

**Methods:**

Fifty healthy adult volunteers (25 males and 25 females, mean age 31.6 ± 3.5 years) were included in this study. Images of the plantar fascia from 100 ft were acquired with ultrasonography, CT and MRI. Ultrasound was used to measure the thickness of the plantar fascia. Imaging data from CT and MRI in a DICOM format were transformed into the Materialise Mimics Innovation Suite 16.0 software for digital analysis. SPSS software (SPSS, USA) was used for statistical analysis. The reliability was established by a t-test. Moreover, 42 patients with unilateral plantar fasciitis were examined by ultrasonography.

**Results:**

There were no significant differences between the three imaging modalities for patients of the same sex (*P* > 0.05). There were no statistically significant differences between the left and right sides for patients of the same sex (*P* > 0.05), but the difference between males and females was statistically significant (*P* < 0.01). There were no significant differences between US, CT and MRI in the normal group, but there were significant differences in the plantar fasciitis group evaluated with ultrasound. The plantar fascii of normal male subjects are significantly thicker than those of the normal female.

**Conclusion:**

Ultrasonography can be a relatively simple and reliable method for the measurement of plantar fascia thickness.

## Background

The plantar fascia is part of the fascia of the foot; the plantar fascia starts from the calcaneal tubercle, has a triangular shape and extends towards the metatarsal bones. One of the important roles of the plantar fascia is to maintain the arch of the foot. In total, 80% of calcaneodynia cases are caused by metatarsal injury [1]. The diagnosis of metatarsal membrane injury is difficult in the clinic because of subjectivity and uncertainty in evaluating the factors. Because of its superficiality, shape and lack of density contrast, examinations of the plantar fascia has been limited in previous research. With the development of imaging technology and progress in equipment, an increasing number of imaging methods have been applied to the inspection of the plantar fascia. Different methods of imaging the plantar fascia have been shown, and ordinary radiography, ultrasound and MRI provide valuable information [2]. Osborne and others have suggested that the thickening of the plantar fascia, the appearance of an abnormal fat pad under the plantar fascia and changes of the bone cortex constitute the base imaging features of plantar fasciitis [3]; the diagnostic value of ultrasound for plantar fasciitis has been confirmed by many scholars [4–8], but the reliability is still controversial [9]. Although there are no significant differences in the examination of the thickness of the plantar fascia between ultrasonography and MRI, MRI is considered the most sensitive method for diagnosing plantar fasciitis [10].

The plantar fascia is a major arch support structure of the feet; the plantar fascia is a fibrous tendon that maintains the longitudinal arch of the foot and starts at the calcaneal tubercle and ends at the metatarsals [11]. During walking, the load on the plantar fascia exceeds its capacity, which results in a degenerative change or plantar fascia injury that causes inflammatory pain [12–14]. Plantar fasciitis is the most common cause of pain. Therefore, the study of the plantar fascia is very important for the diagnosis and mechanical analysis of plantar fasciitis.

The aim was to establish the reliability of ultrasound for the evaluation of the plantar fascia by comparing normal ultrasound, CT and MRI measurements of the normal plantar fascia in orthogonal planes and to evaluate the use of ultrasound in patients with plantar fasciitis.

## Methods

### Selection of studies

A total of 50 healthy adult volunteers (25 males, 25 females) with a mean age of 31.6 years (ranging from 18 to 51 years) were enrolled in this study. The height ranged from 155 to 181 cm, with an average of 172.3 ± 7.6 cm; body weight ranged from 49 to 90 kg, with an average of 78.3 ± 3.6 kg. All data fit a normal distribution. The exclusion criteria for this study were subjects who had flat feet, tumours or severe bone deformities. All experiments were performed in a double-blind manner. The subject was approved by the medical ethics committee of the Inner Mongolia Medical University (No.YKD2017156), and all patients have signed an informed consent.

### Ultrasound scans

**Fig. 1:**
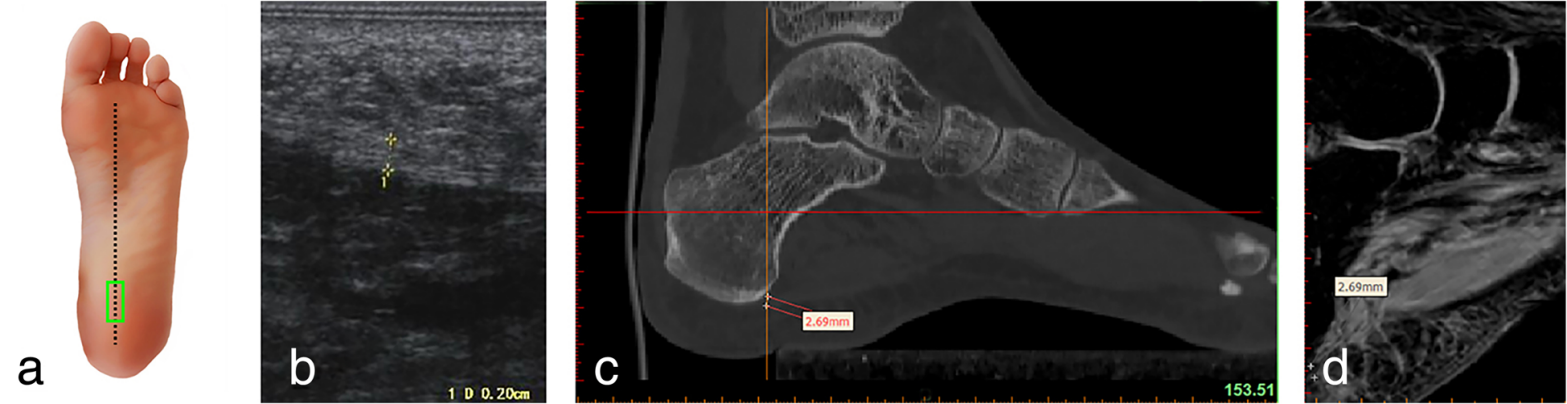
Ultrasound, CT, and MRI measurements of the thickness of the plantar fascia at its origin (longitudinal section) **a** Ultrasonic probe is placed (green panel). **b** Ultrasonic measurement. **c** Measuring the thickness of the plantar fascia econstruction CT images. **d** Measuring the thickness of the plantar fascia reconstruction MRI images

**Fig. 2:**
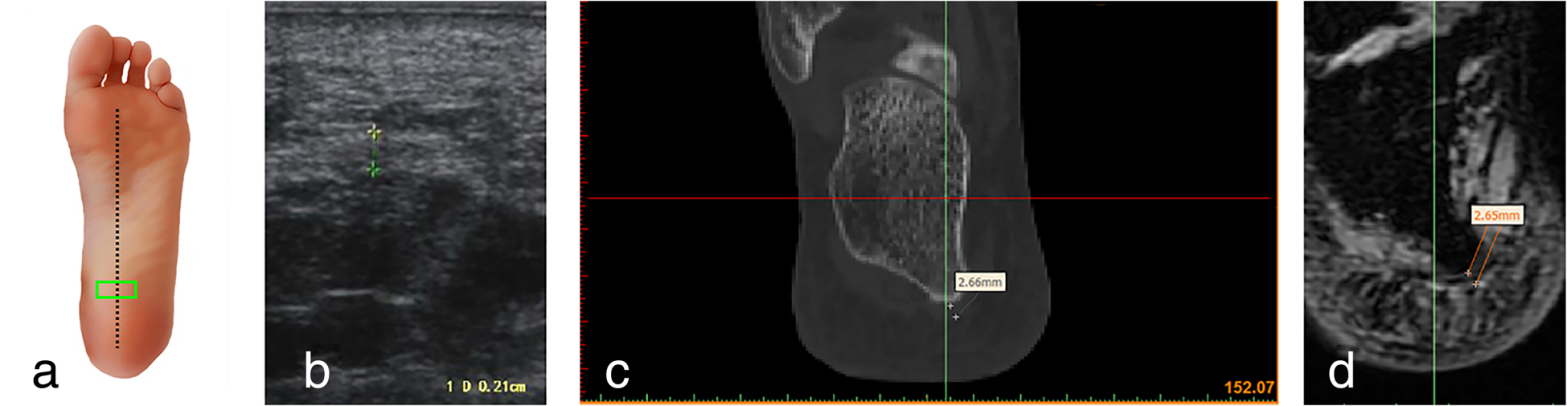
Ultrasound, CT and MRI measurements of the thickness of the plantar fascia at its origin (transverse section) **a** Ultrasonic probe is placed (green panel). **b** Ultrasonic measurement. **c** Measuring the thickness of the plantar fascia reconstruction CT images. **d** Measuring the thickness of the plantar fascia reconstruction MRI images

**Fig. 3:**
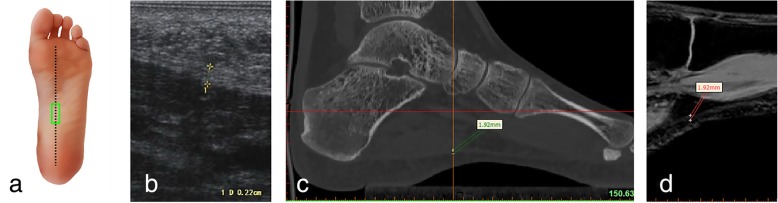
Ultrasound, CT and MRI measurements of the arch of the plantar fascia (longitudinal section) **a** Ultrasonic probe is placed (green panel). **b** Ultrasonic measurement. **c** Measuring the thickness of the plantar fascia reconstruction CT images. **d** Measuring the thickness of the plantar fascia reconstruction MRI images

**Fig. 4:**
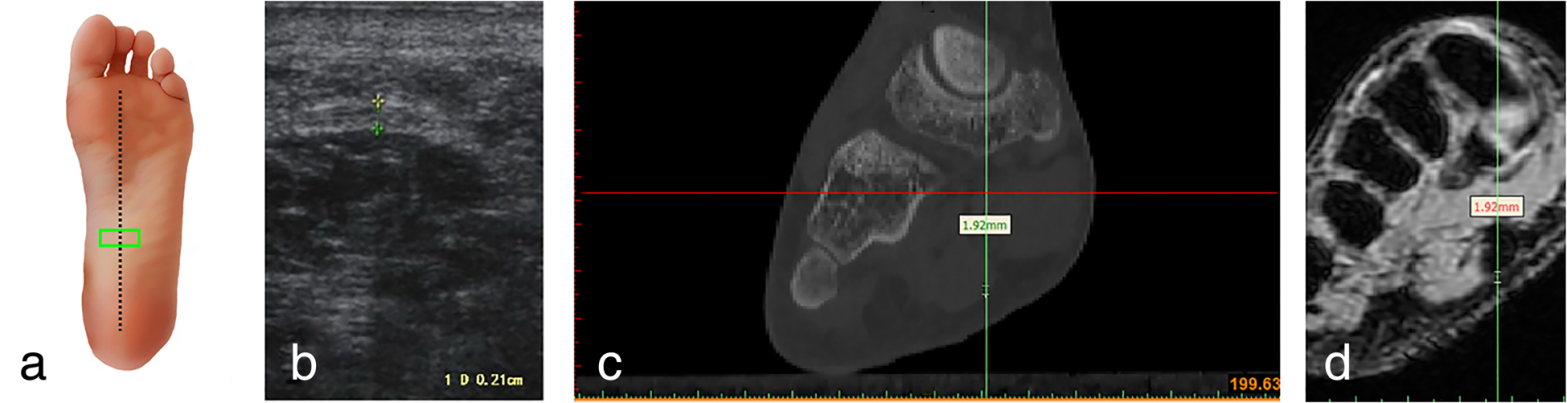
Ultrasound, CT and MRI measurements of the arch of the plantar fascia (transverse section) **a** Ultrasonic probe is placed (green panel). **b** Ultrasonic measurement. **c** Measuring the thickness of the plantar fascia reconstruction CT images. **d** Measuring the thickness of the plantar fascia reconstruction MRI images

**Fig. 5:**
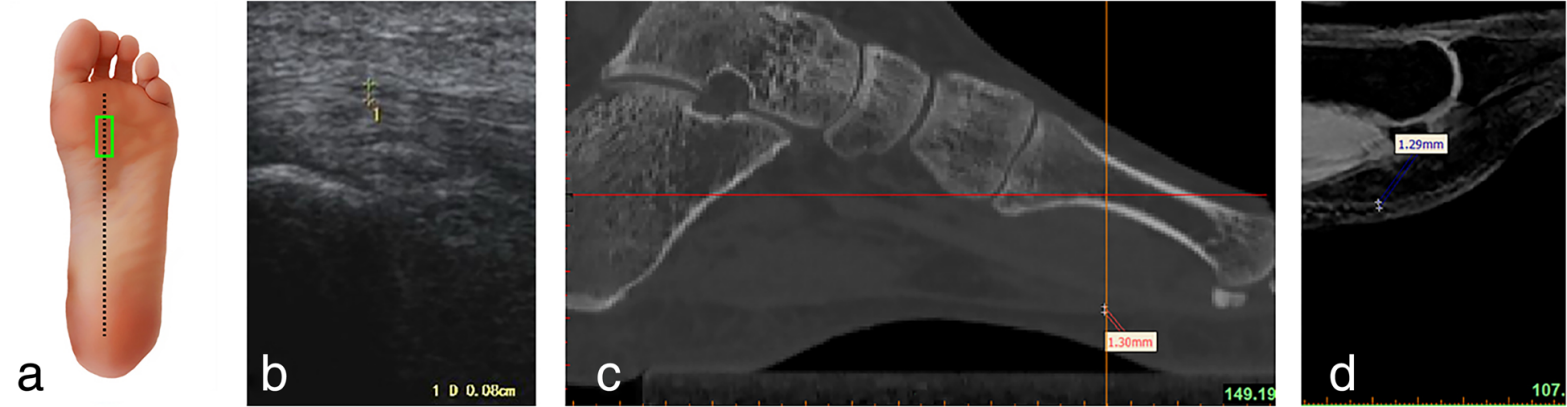
Ultrasound, CT and MRI measurements of the thickness of the plantar fascia between the first and second toes (longitudinal section) **a** Ultrasonic probe is placed (green panel). **b** Ultrasonic measurement. **c** Measuring the thickness of the plantar fascia reconstruction CT images. **d** Measuring the thickness of the plantar fascia reconstruction MRI images

**Fig. 6:**
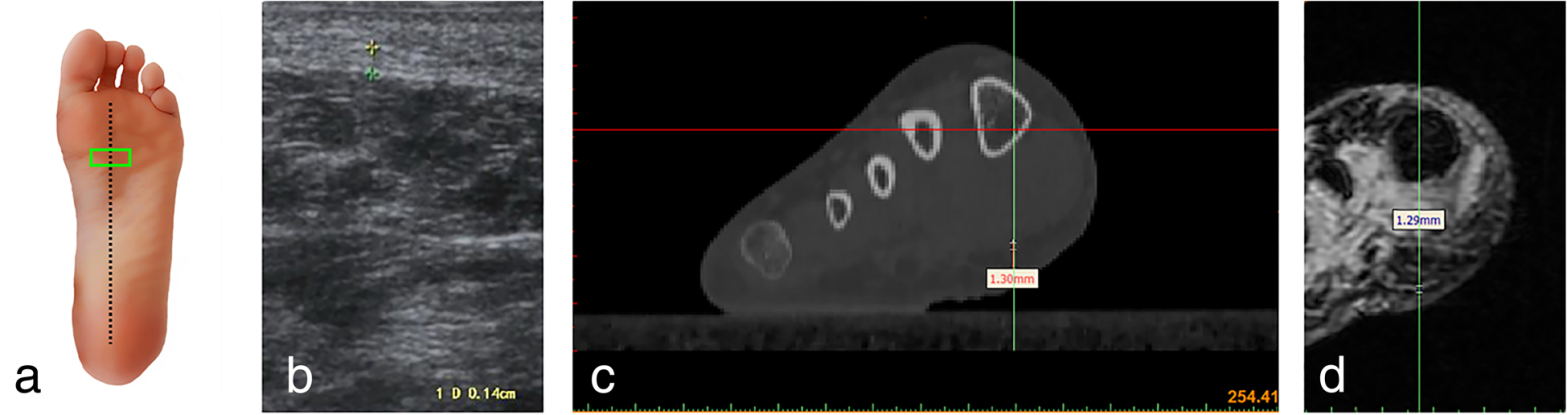
Ultrasound, CT and MRI measurements of the thickness of the plantar fascia between the first and second toes (transverse section) **a** Ultrasonic probe is placed (green panel). **b** Ultrasonic measurement. **c** Measuring the thickness of the plantar fascia reconstruction CT images. **d** Measuring the thickness of the plantar fascia reconstruction MRI images

Measurements of the plantar fascia thickness were taken using HIVISION Preirus ultrasound systems (Hitachi Ltd., Japan) with a linear 5 to 13 MHz transducer. The patient was asked to lie in a prone position with the bilateral lower limbs in full extension and both ankles at 90°. The probe of the ultrasound was placed on a line connecting the second toe and the midheel. The thickness of the plantar fascia was determined at its proximal end, close to the insertion point into the calcaneus, by longitudinal sonograms of the heel [15] (Figs.1a, b and 2a, b); then, readings were also taken at the arch of the foot in the central metatarsal zone (Figs. 3a, b, and 4a, b). Finally, the thickness of the plantar fascia was measured between the base of the first and second toes (Fig. 5a, b and 6a, b). Additionally, the echo changes were observed in two sections, and the thickness and echo of the plantar fascia were observed and measured in the longitudinal section and in the cross-section, respectively. The ultrasound examinations were performed by two physicians, both of whom had rich experience in the diagnosis of musculoskeletal conditions, and the reliability of the repeated measurements between the evaluators was evaluated.

### CT scans

The feet were scanned by a GE Light Speed 64 spiral CT (GE, USA) with the patient in a supine position, the bilateral lower limbs in full extension, and both ankles at 90°; the scan was acquired at 120 kV, a slice thickness of 0.625 mm and a matrix of 512 × 512. The original imaging data were imported into the Materialise Mimics Innovation Suite 16.0 software (Materialise, Belgium) in the DICOM format. The positions for the plantar fascia measurements with CT were the same as those for ultrasound. Three measurements were taken and averaged (Figs. 1c, 2, 3, 4, 5 and 6c).

### MRI scans

All magnetic resonance scans were acquired on a GE Discovery MR 750 3.0 T scanner (GE, USA) using an 8-channel coil, with a slice thickness of 1.0 mm and a matrix of 320 × 224. The patient was in a supine position, and the bilateral lower limbs were fully extended, with the ankle joints at 90°. The scanned images (.dicom files) were processed by the Materialise Mimics Innovation Suite 16.0 software (Materialise, Belgium). Ultrasound was used to analyze and measure from the same site.. Three measurements were taken and averaged (Figs. 1d, 2, 3, 4, 5 and 6d).

### Clinical application

A total of 42 patients with unilateral plantar fasciitis and a clinical history and physical examination highly suggestive of plantar fasciitis were involved in this study at the Affiliated Hospital of Inner Mongolia Medical University. There were 24 women and 18 males; there were 8 patients with bilateral plantar fasciitis, including 3 women and 5 men; the age was 27–65 years (average 47.4 ± 11.4 years), the height was 155–178 cm (average 168.2 ± 6.5 cm), and the weight was 55–80 kg (average 69.7 ± 6.9 kg). The plantar fascia thickness was observed and measured on the longitudinal part of the plantar fascia, the central tendon and the insertion point (at the medial tuberosity of the calcaneus); the thickness of the plantar fascia was recorded.

#### Statistical processing

SPSS v13.0 software (SPSS, Chicago, IL, USA) was used for statistical analysis. The measurement data were first tested by the Shapiro-Wilkes test to determine whether the data were normally distributed. Measurements of the average plantar fascia thickness from ultrasound, CT, and MRI are based on data with a normal distribution and homogeneity of variance. The differences between the average fascia thickness of men and women were compared using two independent sample t-tests, and the left and right sides of the same sex were compared with paired t-tests. Two-tailed *P* values < 0.05 were considered statistically significant.

## Results

In the three normal groups (i.e., ultrasounds, CTs and MRIs of healthy volunteers), there were no significant differences when the thickness of the plantar fascia was measured for patients of the same sex (*P* > 0.05). There were no significant differences between the left and right sides for patients of the same sex (*P* > 0.05). There were no significant differences between the three groups when the measurements acquired from similar positions of the plantar fascia were compared (*P* > 0.05). There were statistically significant differences between males and females. The results of the three tests showed that the average thickness of the plantar fascia was greater in males than in females (*P* < 0.01) (Tables 1, 2 and 3).

**Table 1:** Ultrasound measurements of the average thickness of the plantar fascia (mm)

	Male		Female	
	Left	Right	Left	Right
Origin (LS)	2.88 ± 0.32	2.85 ± 0.30	2.55 ± 0.29	2.52 ± 0.30
Central part (LS)	2.01 ± 0.29	2.02 ± 0.25	1.81 ± 0.22	1.83 ± 0.20
Insertion (LS)	1.30 ± 0.11	1.32 ± 0.12	1.17 ± 0.12	1.15 ± 0.14
Origin (TS)	2.87 ± 0.33	2.86 ± 0.31	2.58 ± 0.31	2.56 ± 0.29
Central part (TS)	2.02 ± 0.28	1.99 ± 0.26	1.81 ± 0.23	1.82 ± 0.22
Insertion (TS)	1.31 ± 0.10	1.33 ± 0.16	1.18 ± 0.11	1.16 ± 0.13

*LS* Longitudinal Section, *TS* Transverse Section

**Table 2:** CT measurements of the average thickness of the plantar fascia (mm)

	Male		Female	
	Left	Right	Left	Right
Origin (LS)	2.86 ± 0.32	2.87 ± 0.31	2.54 ± 0.26	2.55 ± 0.28
Central part (LS)	2.03 ± 0.22	2.02 ± 0.24	1.82 ± 0.20	1.84 ± 0.23
Insertion (LS)	1.33 ± 0.11	1.34 ± 0.13	1.18 ± 0.10	1.16 ± 0.13
Origin (TS)	2.85 ± 0.32	2.84 ± 0.33	2.57 ± 0.30	2.56 ± 0.31
Central part (TS)	2.05 ± 0.23	2.01 ± 0.25	1.81 ± 0.21	1.81 ± 0.24
Insertion (TS)	1.32 ± 0.12	1.35 ± 0.13	1.19 ± 0.12	1.17 ± 0.11

*LS* Longitudinal Section, *TS* Transverse Section

**Table 3:** MRI measurements of the average thickness of the plantar fascia (mm)

	Male		Female	
	Left	Right	Left	Right
Origin (LS)	2.87 ± 0.30	2.86 ± 0.31	2.56 ± 0.28	2.55 ± 0.29
Central part (LS)	2.02 ± 0.29	2.01 ± 0.26	1.82 ± 0.23	1.81 ± 0.19
Insertion (LS)	1.31 ± 0.10	1.29 ± 0.12	1.16 ± 0.11	1.17 ± 0.12
Origin (TS)	2.88 ± 0.31	2.85 ± 0.33	2.59 ± 0.32	2.58 ± 0.33
Central part (TS)	2.05 ± 0.23	2.01 ± 0.25	1.82 ± 0.24	1.83 ± 0.23
Insertion (TS)	1.32 ± 0.12	1.35 ± 0.13	1.20 ± 0.11	1.19 ± 0.12

*LS* Longitudinal Section, *TS* Transverse Section

In the plantar fasciitis groups, in the central part of the plantar fascia, the thickness of the plantar fascia was greater in patients with unilateral plantar fasciitis than in normal volunteers, and the difference was statistically significant (*P* < 0.01). The differences in the thickness of the plantar fascia at its origin were not statistically significant (*P* > 0.05). The thickness of the plantar fascia in patients with plantar fasciitis was greater in males than in females, but the differences were not statistically significant (*P* > 0.05) (Tables 4). Intra-class correlation coefficients (ICC), (95% confidence interval) was used for analyzing inter-rater reproducibility. Six ICC tests were performed on the mean of three examination time measurements for each rater, at three points on both sides. The ICC test showed high inter-rater reproducibility between two raters in all points (ICC > 0.85).

**Table 4:** Comparisons of the mean thickness of the plantar fascia measured by ultrasound in patients with unilateral plantar fasciitis (mm)

	Female (*n* = 24)		Male (*n* = 18)	
	Plantar fasciitis foot	Normal foot	Plantar fasciitis foot	Normal foot
Origin	5.84 ± 1.20	2.55 ± 0.26	5.50 ± 0.73	2.82 ± 0.28
Central part	4.00 ± 1.04	1.82 ± 0.26	3.60 ± 1.45	2.03 ± 0.16
Insertion	1.15 ± 0.14	1.18 ± 0.13	1.27 ± 0.12	1.35 ± 0.15

## Discussion

Hicks et al. [16, 17] proposed the notion that plantar fasciitis was related to an increase in plantar fascia stress, but the research was carried out on cadavers and as such, cannot reflect the normal physiological state of the human body. Through the combination of ultrasound, radiography examinations and stress analysis of plantar fasciitis, Wearing et al. [18, 19] found that the plantar fascia exhibited thickness when plantar fasciitis occurred, causing pain that correlates with the force exerted on the sole of the foot.

In the past, in clinical settings, image techniques were used to rule out potential diseases [20], but imaging had little significance in the diagnosis of plantar fasciitis. At present, the diagnosis of plantar fasciitis mainly depends on the medical history and clinical manifestations but lacks specific means of examination. Some scholars believe that the ultrasound-based diagnosis of plantar fasciitis is an effective, objective, economical and noninvasive method [21].

MRI has good soft tissue resolution and can clearly and accurately show the anatomical structure of the plantar fascia and its pathological changes. MRI can be used to visualize plantar fascia thickening and the area of thickening by estimating the signal enhancement area. MRI can therefore be used to accurately measure and confirm the diagnosis of plantar fasciitis, but due to its cost, its application is limited. Ultrasonography is an effective, objective, inexpensive and noninvasive method for the diagnosis of plantar fasciitis, and there are studies that have suggested that ultrasound can replace MRI in the diagnosis of plantar fasciitis [22], both in China and overseas [21, 23]. The reliability of the measurements of the thickness of the plantar fascia was evaluated by comparing the measurements obtained using an echography machine.

A variety of imaging-based measurements of the plantar fascia in the healthy population were carried out in our research. The data of the plantar fascia were evaluated by digital analysis. The differences between the ultrasound measurement data and the MRI measurement data were not statistically significant and therefore points to the fact that the ultrasound measurements of the plantar fascia thickness are reliable. The data obtained show that the starting point of the plantar fascia is the thickest, while the central and branching parts gradually thin out, which is consistent with the mechanical characteristics of the plantar fascia.

Ozdemir et al. [24] measured the average thickness of the normal plantar fascia and found it to be between 2.2 and 2.5 mm. Gibbon and Long [25] measured the average thickness of the normal plantar fascia and found it to be approximately 3.3 mm. Using a meta-analysis on images of plantar fasciitis, McMillan et al. [26] found that the plantar fascia was significantly thickened during inflammation, and when the thickness was greater than 4 mm, plantar fasciitis could be diagnosed; this diagnostic criterion has been widely recognized. However, none of the above studies analysed the starting, central, or branching points of the plantar fascia. Magnetic resonance imaging can clearly show the plantar soft tissue in both the sagittal and coronal planes. In plantar fasciitis, abnormal MRI signals can not only show the marked thickening of the plantar fascia but can also show the adjacent subcutaneous tissue while showing the signal strength and enhancement point on the calcaneus. Plantar fascia thickening greater than 5 mm, both in ultrasound or MRI, prompts the diagnosis of plantar fasciitis [15, 27–31].

Some scholars [32] have studied the ultrasound-based diagnosis of the distal part of the diseased patellar tendon, which shows low echogenicity on ultrasound, and the interobserver reliability with the use of ultrasound for the diagnosis of patellar tendon disease is high. Song Ye et al. [21] pointed out that when the muscle tissue echogenicity is lower than that of surrounding soft tissue, it is referred to as hypoechoic, but for the plantar fascia, there are no adjacent muscle tissues in its surroundings. This study showed that the ultrasound examination method, the measurement locations and evaluations of the ultrasound images to determine the thickness of the plantar fascia, and the ultrasound images themselves have a high degree of reliability.

There are some limitations in our research. First, the measurements of the ultrasound, MRI, and CT images are subjective, and the measurements are often more pronounced in ultrasound images than in MRI or CT images, depending on the physician’s resolution of the grayscale image. Second, an increased thickness of the plantar fascia is not the only criterion for a diagnosis of plantar fasciitis; changes in the thickness of the plantar fascia is only one of the factors for plantar fasciitis, and the other factors include a decrease in echogenicity and an increase in the blood flow of the fascia, which may cause false-negative or false-positive results. Finally, the number of subjects and patients was relatively small, and the sample size needs to be expanded to further verify the results.

## Conclusion

Through the digital analysis and evaluation of plantar fascia data, it was concluded that there were no statistically significant differences between the ultrasound measurements and the MRI and CT measurements, which indicates hat the ultrasound measurements of the plantar fascia thickness were accurate. In conclusion, the measurements of the thickness of the plantar fascia with ultrasound have a high degree of confidence and may provide evidence-based recommendations to patients with plantar fasciitis.

## Data Availability

The datasets generated and/or analysed during this study are available from the corresponding author upon reasonable request.
